# Clinical and Comorbidity-Related Factors Associated with Health-Related Quality of Life in Patients with Heart Failure: A Cross-Sectional Study

**DOI:** 10.3390/jcm15062376

**Published:** 2026-03-20

**Authors:** Teodora-Gabriela Alexescu, Mara-Ioana Somesfalean, Mirela-Georgiana Perne, Cezara-Andreea Gerdanovics, Ioana Para, Mircea-Vasile Milaciu, Angela Cozma, Codruța-Claudia Gherman-Lencu

**Affiliations:** 1 4th Medical Discipline, Department of Internal Medicine, “Iuliu Hațieganu” University of Medicine and Pharmacy, Republicii Street, No. 18, 400015 Cluj-Napoca, Romania; 2 Faculty of Medicine, “Iuliu Hațieganu” University of Medicine and Pharmacy, 400012 Cluj-Napoca, Romania; 3 Endocrinology-Fundamental Disciplines, Department 1, Faculty of Nursing and Health Sciences, “Iuliu Hațieganu” University of Medicine and Pharmacy, Republicii Street, No. 18, 400015 Cluj-Napoca, Romania

**Keywords:** heart failure, quality of life, key determinants

## Abstract

Heart failure is one of the most prevalent conditions worldwide, exerting a substantial impact on both prognosis and quality of life. It affects both psychological and physical domains, leading to multiple limitations in patients’ daily lives. **Background/Objectives:** The primary objective of this study was to identify clear factors related to disease severity, living conditions, and associated comorbidities that may negatively influence the quality of life of patients with heart failure. **Methods:** This study had a cross-sectional observational design and was conducted between 2024 and 2025 in the Department of Internal Medicine of the CF University Hospital in Cluj-Napoca, Romania. A total of 122 patients (mean age 67.9 ± 9.69 years, 62% women) diagnosed with heart failure, regardless of ejection fraction, and hospitalized in the Internal Medicine department during the study period were included. All participants self-administered the Minnesota Living with Heart Failure Questionnaire. **Results:** The analysis identified several factors associated with better quality of life, including female sex, atrial fibrillation with electrostimulated heart rhythm, type 2 diabetes mellitus, arterial hypertension, hepatitis, and chronic gastritis. Conversely, several factors were found to have a negative association with quality of life, such as advanced age, obesity, higher New York Heart Association (NYHA) functional class, reduced ejection fraction, valvular heart disease, obstructive sleep apnea syndrome, cervical–thoracic–lumbar polydiscopathy, hyperthyroidism, and hepatic steatosis. **Conclusions:** These results highlight the importance of key factors associated with quality of life in patients with heart failure.

## 1. Introduction

Heart failure is a complex clinical syndrome associated with structural and/or functional cardiac abnormalities resulting from conditions that impair the heart’s pumping function [1,2]. It is characterized by a clinical syndrome comprising symptoms such as dyspnea, asthenia, and fatigue, as well as objective signs including peripheral edema, pulmonary crackles, elevated jugular venous pressure, and hepatomegaly [3,4]. According to an observational study including 23,341 patients, the leading cause of heart failure was ischemic heart disease (38.1%), followed by arterial hypertension (20.2%) and non-ischemic dilated cardiomyopathy (15.4%) [5].

It represents a major global public health problem, with a recently estimated prevalence of over 64 million individuals worldwide. This number continues to rise, primarily due to population aging, improved survival following acute cardiovascular events, and the increasing prevalence of risk factors such as hypertension, diabetes, and obesity [6]. The incidence of heart failure has remained stable or slightly decreased in high-income countries; however, the absolute number of cases continues to increase, reflecting ongoing demographic and epidemiological changes [7].

In Western countries, heart failure affects approximately 1–2% of the general population, with prevalence increasing markedly with age, reaching 8.4% among individuals older than 75 years [8]. Men are more likely to develop heart failure than women and tend to do so at a younger age [9].

Quality of life is defined as an individual’s subjective perception of his or her health status across multiple domains, including physical and emotional well-being, social functioning, economic conditions, and environmental factors. In heart failure, the concept of health-related quality of life (HRQoL) has major clinical relevance, as it reflects the impact of the disease on daily activities, interpersonal relationships, and emotional status [10]. Assessment of quality of life is commonly performed using validated instruments, either generic tools such as the SF-36 or disease-specific questionnaires, including the Chronic Heart Failure Questionnaire (CHFQ), the Kansas City Cardiomyopathy Questionnaire (KCCQ), and the Minnesota Living with Heart Failure Questionnaire (MLHFQ) [11,12].

Quality of life is strongly influenced by a variety of clinical and individual factors, which may vary considerably between patients. Age has been shown to affect quality of life, with some studies reporting lower HRQoL among younger patients [13], while others have demonstrated the opposite trend [14]. Regarding sex differences, although women generally exhibit a more favorable clinical course, they have been reported to have poorer quality of life as assessed by the Kansas City Cardiomyopathy Questionnaire (KCCQ) [15], the MLHFQ, and the EQ-5D-5L [16]. In contrast, a study published in the International Journal of Cardiology found no significant sex-related differences in quality of life when assessed using the MLHFQ [17].

According to the study conducted by Beneš J. and colleagues [18], an increased number of comorbidities associated with heart failure did not lead to a deterioration in quality of life as measured by the MLHFQ. However, markers of disease severity, such as New York Heart Association (NYHA) functional class, systolic arterial hypertension, and the required dose of furosemide, were found to be negatively associated with quality of life. Findings from a 2020 scoping review demonstrated a 60.8-fold reduction in quality of life among patients with depression (*p* < 0.001). Additionally, patients with anemia and iron deficiency were 2.2 times more likely to experience impaired quality of life (*p* = 0.002), while those with isolated iron deficiency were 1.6 times more likely to have reduced quality of life (*p* = 0.025) [19]. Elevated body mass index (BMI) has also been identified as a negative determinant of quality of life, with significant differences demonstrated using both the MLHFQ and KCCQ instruments [20,21].

Obstructive sleep apnea syndrome (OSAS) has a higher prevalence among patients with heart failure, particularly in those with reduced ejection fraction (LVEF < 40%) [22,23]. A systematic review assessing the impact of obstructive sleep apnea on quality of life in patients with heart failure demonstrated poorer quality of life, worsening cardiac function, and reduced physical capacity compared with patients without sleep-disordered breathing [24].

Lower quality of life has been reported in younger patients (<40 years), who, despite having a better functional profile according to NYHA classification, exhibited higher MLHFQ scores [13]. Conversely, a recent study demonstrated a decline in quality of life with advancing age, particularly affecting the emotional subscale of the MLHFQ [25].

Recent therapeutic advances in heart failure management have also demonstrated meaningful improvements in health-related quality of life [26,27]. Sodium–glucose cotransporter-2 (SGLT2) inhibitors, including empagliflozin and dapagliflozin, have consistently shown beneficial effects not only on cardiovascular outcomes but also on patient-reported quality-of-life measures in heart failure populations across the ejection fraction spectrum [28]. Sodium–glucose cotransporter-2 (SGLT-2) inhibitors have been shown to significantly improve quality of life as assessed by the KCCQ, with a mean increase of 2.05 points in the total score [29]. Other studies have confirmed similar improvements in KCCQ scores [30,31], which were maintained regardless of sex, ejection fraction, or the presence or absence of diabetes mellitus [28]. The PARADIGM-HF trial demonstrated the superiority of sacubitril/valsartan (ARNI) over enalapril in improving quality of life among patients with heart failure with reduced ejection fraction. Results showed a mean increase in KCCQ-CS of +0.64 in the ARNI group and a decrease of −0.29 in the enalapril group compared with baseline. Similar findings were observed for the KCCQ-OS (+1.13 versus −0.14) [32]. These benefits were also maintained in the PARASAIL study [33]. In addition, emerging therapies targeting the nitric oxide–soluble guanylate cyclase pathway, such as vericiguat, have been proposed as promising options for patients with worsening heart failure, with potential benefits extending to symptom burden and functional status [34]. These therapeutic advances highlight the growing importance of integrating patient-reported outcomes, including health-related quality of life, into the clinical evaluation and management of heart failure.

## 2. Materials and Methods

This cross-sectional observational study was conducted between August 2024 and January 2025 in the Department of Internal Medicine of the CF University Hospital in Cluj-Napoca, Romania. Data were collected from a sample of 122 patients who met the following inclusion criteria: at the time of questionnaire completion, participants had a documented diagnosis of heart failure according to existing medical records and were hospitalized at the CF University Hospital in Cluj-Napoca during the study period. The sole exclusion criterion was refusal to participate in the study. Patients were consecutively enrolled during the study period in order to minimize potential selection bias and to reflect the real-world clinical population treated in our center. The study population consisted of hospitalized patients with heart failure evaluated during admission, most of whom were admitted for acute decompensated heart failure or worsening heart failure symptoms. No formal sample size calculation was performed prior to study initiation, as the study was designed as an exploratory observational analysis conducted within a predefined time frame. Prior to study initiation, approval for study conduct was obtained from the Ethics Committee of the CF Clinical Hospital in Cluj-Napoca. All enrolled patients were administered the Minnesota Living with Heart Failure Questionnaire, a 21-item instrument designed to assess the impact of heart failure–related symptoms on patients’ daily lives and overall quality of life. The Minnesota Living with Heart Failure Questionnaire was used with permission for student project purposes, the study was conducted as part of a bachelor’s thesis.

General patient data were recorded, including details related to heart failure (NYHA functional class, ejection fraction, and type of diastolic dysfunction), associated comorbidities, living and working conditions, toxic substance use, and activity level. Physical activity level was assessed indirectly, based on patient-reported exercise tolerance and the average daily duration of physical activities. Physical activity was assessed based on self-reported habitual activity levels obtained during the clinical interview at the time of hospitalization. Patients were asked to estimate their usual level of physical activity, and this information was used to construct a general physical activity index reflecting sedentary, moderate, or higher levels of daily activity.

### Statistical Analysis

This study had a cross-sectional observational design, and statistical analysis was performed using Jamovi software, version 2.6.44. Additional graphical representations were generated using Microsoft Excel (version 2509). Descriptive statistics were calculated using mean, standard deviation, median, interquartile range, percentages, and histograms to characterize the study population. Normality of continuous variables was assessed using the Shapiro–Wilk test, and based on the results, either parametric or non-parametric tests were applied.

To compare MLHFQ scores between groups and to evaluate the influence of clinical and occupational variables on quality of life, multiple linear regression analysis was performed.

Results were illustrated using a coefficient plot. Additionally, the Mann–Whitney U test was applied for non-normally distributed variables when comparing MLHFQ scores between two groups, while the Kruskal–Wallis test was used for comparisons involving three or more groups. Spearman’s rank correlation coefficient was used to assess relationships between continuous variables (age, BMI, physical activity index) and MLHFQ score. Statistical significance was set at *p* < 0.05.

The multivariable regression model included clinically relevant variables selected based on prior literature and results of univariable analyses. Given the relatively limited sample size, the number of predictors included in the final model was restricted in order to reduce the risk of model overfitting and to maintain statistical stability. Model diagnostics were performed to verify the assumptions of linear regression, including evaluation of residual distribution and homoscedasticity. Unstandardized regression coefficients were reported to facilitate the clinical interpretation of the results. Although some variables showed deviations from normal distribution, linear regression analysis was applied due to its robustness to moderate violations of normality assumptions. Model diagnostics were performed to assess the validity of regression assumptions, including evaluation of residual distribution and homoscedasticity.

## 3. Results

The study included a total of 122 patients, of whom 62% were women and 38% were men. The median age of the study population was 69 [59, 74] years. Among women, the median age was slightly higher at 69 [60, 74] years compared with men at 68 [59, 75] years; however, no statistically significant difference was observed between the two groups (*p* = 0.81, Mann–Whitney U test).

Variation in systolic function distribution across age groups was assessed using the Kruskal–Wallis test (χ^2^ = 23.2, *p* < 0.01). Post hoc analysis and median comparisons showed that patients with mildly reduced left ventricular ejection fraction (LVEF) (Mdn = 78) were significantly older than both patients with preserved LVEF (Mdn = 68; *p* < 0.01) and those with reduced LVEF (Mdn = 59; *p* < 0.01) (Table 1).

The majority of patients were classified as NYHA functional class II (59%) and III (39.3%), while NYHA class IV accounted for only 1.6% of cases and included exclusively female patients. Preserved systolic function (LVEF > 50%) was observed in 72.1% of the study population, whereas 14.8% had reduced systolic function (LVEF < 40%) and 13.1% had mildly reduced systolic function (LVEF 41–50%).

Most patients were in sinus rhythm (85.2%), 9.8% presented with atrial fibrillation, and the remaining 4.9% had a paced cardiac rhythm. Regarding smoking status, 65.6% of participants identified as non-smokers, while the remainder were either current smokers (16.4%) or former smokers (18%). The median body mass index (BMI) of the study cohort was 29.4 [27.8–29.4].

Based on the assessment of physical activity level, 4.9% of patients were classified as physically active, 50.8% as moderately active, and 44.3% as having a low level of physical activity.

**Figure 1:**
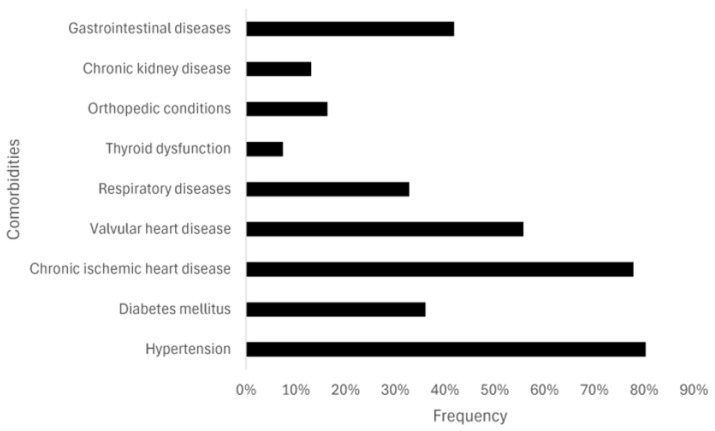
Frequency of comorbidities in the studied population. The figure illustrates the prevalence of major clinical comorbidities among the study participants, expressed as percentages of the total cohort.

The distribution of comorbidities within the study population is illustrated in Figure 1. Among gastrointestinal conditions, hepatic steatosis was the most prevalent comorbidity (17.2%), followed by gastroesophageal reflux disease (GERD) (13.1%). Chronic obstructive pulmonary disease (COPD) was identified in 14.8% of patients, including GOLD stage 2 in 8.2% and GOLD stage 3 in 6.6%. Orthopedic conditions were observed at relatively similar proportions, including gonarthrosis and polydiscopathy (each 4.9%) and spondylarthrosis (6.6%) (Figure 1). Grade II arterial hypertension was present in 55.7% of the study participants.

Normality of data distribution was assessed using the Shapiro–Wilk test. The MLHFQ total score and the MLHFQ physical subscore showed a normal distribution (*p* = 0.312 for the total score and *p* = 0.079 for the physical subscore), whereas the MLHFQ emotional subscore was not normally distributed (*p* < 0.001) (Table 2).

Following the multiple linear regression analysis performed in the present study, female sex was associated with lower values for both the MLHFQ total score and the physical subscore, corresponding to a higher quality of life. With regard to age, the analysis showed that the MLHFQ physical subscore increased by 0.152 points for each additional year of age, indicating poorer quality of life among older patients (*p* = 0.049). A significant association was also observed between body mass index (BMI) and the MLHFQ total score, with an increase of 0.4438 points in the score for each additional BMI unit.

A progressive decline in quality of life was observed with increasing New York Heart Association (NYHA) functional class, affecting both the MLHFQ total score and the physical subscore. Patients with mildly reduced ejection fraction (LVEF 41–50%) reported poorer quality of life than those with preserved ejection fraction (LVEF > 50%), as reflected by higher MLHFQ scores. No significant differences were observed in patients with reduced systolic function (LVEF < 40%).

**Figure 2:**
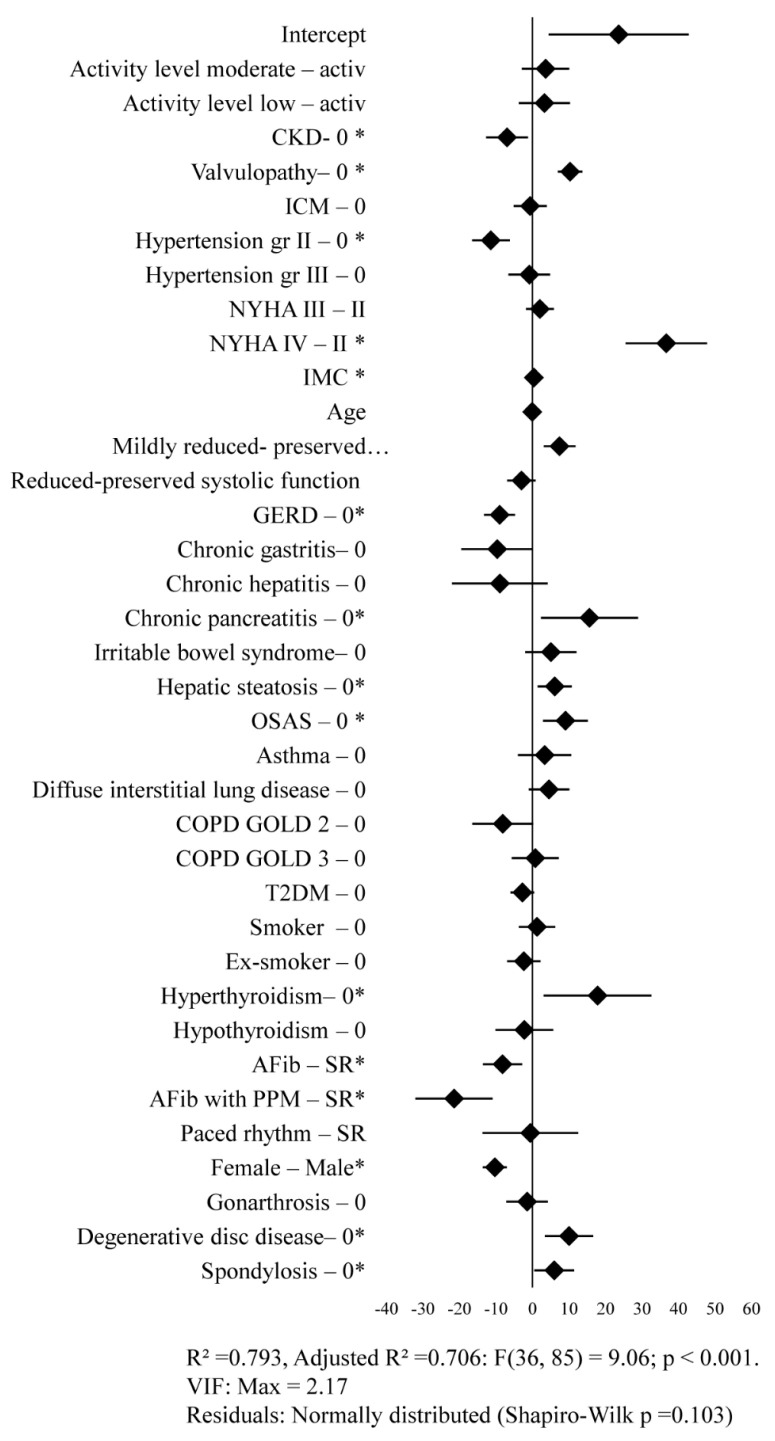
Impact of clinical factors on the MLHFQ total score. The plot displays the regression coefficients (β) from a multivariable linear regression model. Each diamond represents the adjusted effect estimate for the corresponding predictor (difference in MLHFQ total score relative to the reference category, where applicable), and horizontal lines indicate 95% confidence intervals. The vertical line at β = 0 denotes no association. Potential predictors marked with an asterisk are statistically significant in the model. Model performance metrics (R^2^, adjusted R^2^, F-test), multicollinearity assessment.

Several of the investigated comorbidities were associated with poorer quality of life among the study population. Patients with valvular heart disease had significantly higher mean MLHFQ scores, with a difference of 10.35 points for the total score (*p* < 0.001) and 3.727 points for the physical subscore compared with those without valvular disease. Higher mean MLHFQ total scores, indicating lower quality of life, were also observed in patients with alcohol- and tobacco-related pancreatitis, hepatic steatosis, obstructive sleep apnea syndrome (OSAS), and hyperthyroidism. Among these conditions, only OSAS was also associated with impairment of the physical subscore, with a mean increase of 6.828 points (*p* = 0.003). In the analysis of orthopedic comorbidities, patients with cervical–thoracic–lumbar polydiscopathy exhibited significantly higher MLHFQ total scores (+10.049 points, *p* = 0.003) as well as higher physical subscores (+9.483 points, *p* < 0.001) compared with patients without orthopedic pathology. In contrast, patients with spondylarthrosis showed a significant difference only with respect to the MLHFQ total score (Figure 2).

**Figure 3:**
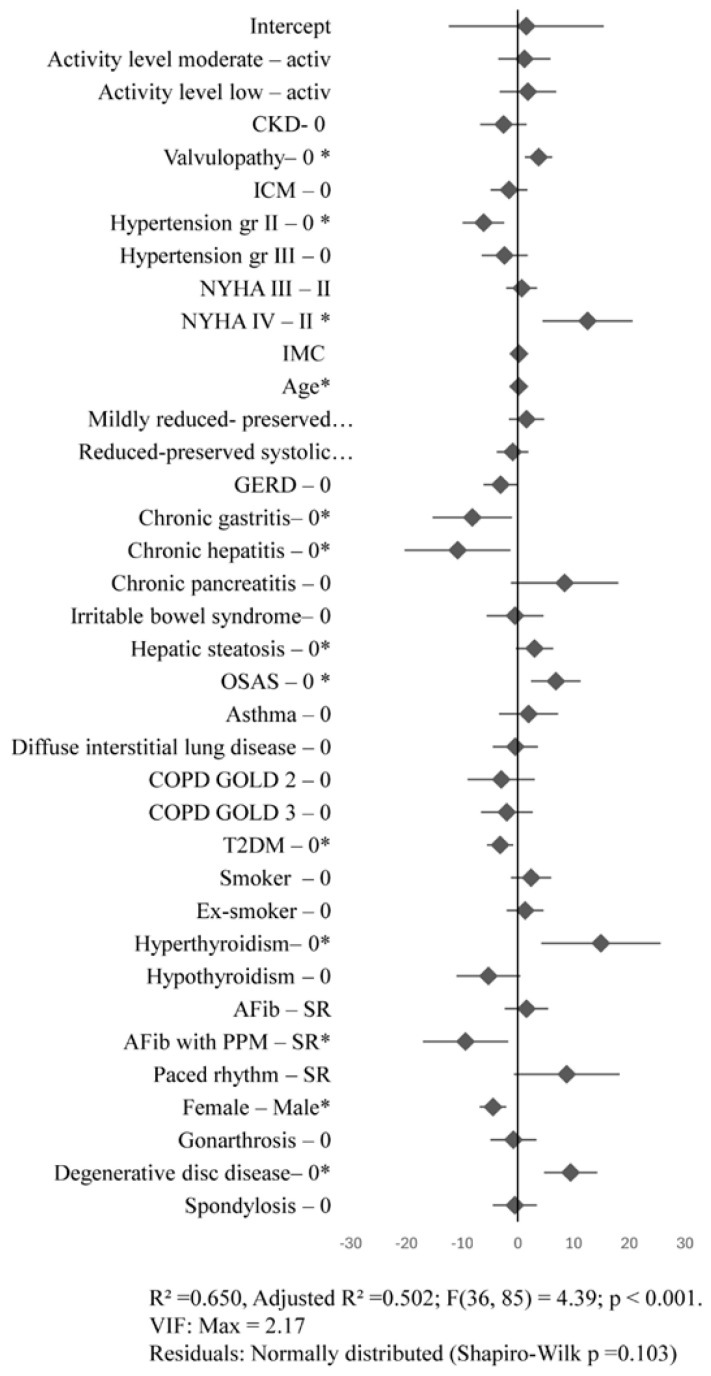
Influence of clinical factors on the MLHFQ physical subscore. Points represent the estimated B coefficients, while horizontal lines indicate the corresponding 95% confidence intervals. The vertical line at zero denotes the absence of an effect; overlap of the confidence interval with this line indicates a lack of statistical significance. The model was simultaneously adjusted for all variables included in the analysis. * *p* < 0.05. Coding: 0 = absence of comorbidity/risk factor; CKD = chronic kidney disease; CIC = chronic ischemic cardiomyopathy; HTN = arterial hypertension; DM = diabetes mellitus; GERD = gastroesophageal reflux disease; PM = electrostimulated cardiac rhythm; AF = atrial fibrillation; SR = sinus rhythm.

Conversely, certain conditions were associated with better quality of life, reflected by lower MLHFQ scores. Grade II arterial hypertension and atrial fibrillation with electrostimulated cardiac rhythm were associated with improved quality of life for both the MLHFQ total score and the physical subscore. Other conditions were associated with improved quality of life either according to the MLHFQ total score, such as gastroesophageal reflux disease and chronic kidney disease, or according to the MLHFQ physical subscore, including type 2 diabetes mellitus, hepatitis, and chronic gastritis (Figure 3).

To identify subjects with a disproportionate influence on the regression model, Cook’s distance was calculated, yielding a maximum value of 0.605 for the MLHFQ total score and 0.0881 for the physical subscore. Both values were below the threshold of 1 recommended in the literature, indicating no influential outliers.

Multiple linear regression was also applied to the MLHFQ emotional subscore; however, the Shapiro–Wilk normality test performed on the model residuals demonstrated a significant deviation from normal distribution (*p* < 0.001). This finding was further supported by inspection of the Q–Q plot, thereby limiting the statistical validity of the regression model for this outcome. Consequently, the Mann–Whitney U test was used to compare the distribution of the MLHFQ emotional subscore between two groups.

Comparative analysis revealed that only patients with diabetes mellitus exhibited a significantly higher quality of life compared with those without this condition, reflected by a substantially lower median MLHFQ emotional subscore (4.50 vs. 7; W = 1318, *p* = 0.033) (Table 3).

Furthermore, the Kruskal–Wallis test was used to compare differences in the MLHFQ emotional subscore according to NYHA functional class, left ventricular ejection fraction, and arterial hypertension grade (Table 4).

Post hoc analysis using the Dwass–Steel–Critchlow–Fligner test revealed that the presence of grade II arterial hypertension was associated with a significantly higher quality of life. Accordingly, patients with this comorbidity exhibited substantially lower median MLHFQ emotional subscore values compared with those without arterial hypertension (5.50 vs. 8.00, *p* = 0.015). A similar trend was observed with respect to physical activity level, with patients reporting a moderate activity level demonstrating significantly lower MLHFQ emotional scores compared with those with low activity levels (median = 6 vs. 7, *p* = 0.037).

Spearman’s rank correlation was applied to assess the relationship between age, physical activity index, body mass index (BMI), and the MLHFQ emotional subscore; however, no statistically significant correlations were identified.

## 4. Discussion

Our analysis demonstrated a heterogeneous impact of comorbidities on quality of life, with findings largely consistent with previously published data.

The negative influence of advanced age on quality of life represents a discordant finding compared with certain previously cited studies [17]; however, it is consistent with other reports demonstrating poorer quality of life among older patients, as assessed using the MLHFQ [35], KCCQ [36], and EQ-5D instruments [14]. In the present study, female patients exhibited a higher quality of life compared with male patients. This observation adds to the previously reported, somewhat contradictory evidence in the literature. While some studies have reported poorer quality of life among women [18,37], others found no significant sex-related differences when using the MLHFQ [17]. The findings of the present study may be partly explained by the higher prevalence of reduced ejection fraction among male patients.

Patients with mildly reduced systolic function (LVEF 41–50%) reported poorer quality of life compared with those with preserved systolic function (LVEF > 50%). Although this finding contrasts with some previously reported data [37], it aligns with other studies suggesting a progressive decline in quality of life with decreasing ejection fraction [38]. Several factors may explain this observation. Patients with HFmrEF may experience a higher symptomatic burden, including exercise intolerance and functional limitations, which may negatively influence patient-reported outcomes. In addition, the classification of heart failure phenotypes based solely on left ventricular ejection fraction may lead to a degree of overlap or misclassification, as LVEF is a dynamic parameter that may change over time depending on disease progression and treatment response. Furthermore, differences in therapeutic strategies and treatment optimization between HFpEF and HFmrEF populations may also influence symptom perception and quality-of-life outcomes.

Symptom burden remains one of the most important factors associated with quality of life in patients with heart failure. Among the classical manifestations of heart failure, dyspnea remains the cardinal clinical symptom and one of the main drivers of functional limitation and reduced quality of life. The severity of dyspnea, particularly during physical exertion, as well as recurrent episodes of decompensation, may substantially influence patients’ daily functioning and perceived health status. In acute and chronic heart failure, pulmonary congestion and elevated left-sided filling pressures contribute to impaired gas exchange, increased work of breathing, and activation of neurohormonal pathways, further worsening symptom burden and functional capacity [39].

Furthermore, the interaction between respiratory mechanics and cardiac function plays a critical role in shaping clinical symptoms. Changes in intrathoracic pressure, pulmonary congestion, and ventilation–perfusion mismatch can significantly affect cardiac preload, afterload, and stroke volume, thereby influencing both hemodynamic status and perceived symptom severity in patients with heart failure [40].

These mechanisms highlight how symptom burden—particularly dyspnea severity and recurrent decompensation episodes—may interact with comorbidities and contribute to variations in MLHFQ scores observed in clinical cohorts. Therefore, the evaluation of HRQoL should always be interpreted in the broader clinical context of heart failure severity, symptom burden, and cardiorespiratory interactions. Regarding negative factors, the presence of obesity, valvular heart disease, obstructive sleep apnea syndrome (OSAS), polydiscopathy, hyperthyroidism, and hepatic steatosis was associated with a significant reduction in quality of life. These findings corroborate existing evidence, particularly with respect to obesity [21] and OSAS [24,41].

Interestingly, several comorbidities—including atrial fibrillation with electrostimulated cardiac rhythm, type 2 diabetes mellitus, arterial hypertension, hepatitis, and chronic gastritis—were associated with a positive impact on quality of life in our cohort. These results contrast with studies reporting impaired quality of life among patients with diabetes mellitus [42,43,44] and arterial hypertension [45,46]. Such findings may be partly explained by effective therapeutic management or by psychological adaptation mechanisms, whereby patients recalibrate their health-related expectations. Additionally, interventions such as pacemaker implantation in patients with atrial fibrillation have previously been associated with improved quality of life outcomes [47]. Recent literature has increasingly emphasized the impact of rhythm-control strategies and device-based therapies on patient-reported outcomes in cardiovascular disease. Catheter ablation, currently considered a cornerstone rhythm-control therapy in atrial fibrillation, has been associated with significant improvements in symptoms, functional capacity, and health-related quality of life. These benefits are largely related to the reduction in arrhythmia burden and the restoration of a more stable cardiac rhythm, which may translate into improved daily functioning and patient well-being [48]. In addition, cardiac implantable electronic devices (CIEDs), including pacemakers and other rhythm-management systems, represent an essential component of modern arrhythmia management. These devices can improve rhythm stability and hemodynamic performance, potentially reducing symptom burden and enhancing overall clinical status in selected patients. The increasing use of CIEDs in contemporary cardiology reflects their important role in improving patient outcomes and supporting long-term disease management [49]. Taken together, these observations may partly explain the association observed in our cohort between atrial fibrillation with electrostimulated cardiac rhythm and improved quality-of-life scores. Rhythm stabilization achieved through device therapy or rhythm-control strategies may contribute to better symptom control and improved patient perception of health status.

Some of the observed associations between specific comorbidities and higher quality-of-life scores may appear counterintuitive. Several methodological and clinical factors may explain these findings. First, certain conditions such as arterial hypertension or type 2 diabetes mellitus may have been well controlled at the time of evaluation, thereby exerting a limited symptomatic burden. Second, patients with these chronic conditions are often subject to closer medical monitoring and more frequent therapeutic optimization, which may contribute to improved overall disease management and perceived health status. Additionally, differences in disease severity across patients, together with the relatively small sample size and the monocentric design of the study, may have introduced a degree of selection bias. Finally, although multicollinearity between potential predictors was assessed using variance inflation factor analysis and no significant issues were detected, potential interactions between comorbidities cannot be completely excluded and may have influenced the regression estimates.

Also, in recent years, the management of heart failure has increasingly emphasized the importance of patient-reported outcomes, including health-related quality of life. Several contemporary pharmacological therapies have demonstrated benefits in improving symptoms and quality-of-life scores in patients with heart failure. For example, SGLT2 inhibitors such as empagliflozin and dapagliflozin have been associated with improvements in functional status and patient-reported outcomes across the spectrum of ejection fraction. In addition, therapies targeting the nitric oxide–soluble guanylate cyclase pathway, including vericiguat, have shown beneficial effects in patients with worsening heart failure. These therapeutic advances highlight the growing importance of health-related quality of life as a clinically relevant outcome and support the need to better understand the clinical factors that may influence patients’ perceived health status. An important limitation of the present study is its monocentric and cross-sectional design, which may restrict the generalizability of the findings to broader populations. Another relevant consideration is the unbalanced sample distribution, characterized by a predominance of female and elderly patients. Assessment of quality of life using self-reported questionnaires, although widely employed in clinical research, inherently introduces subjectivity and interindividual variability. Moreover, the MLHFQ primarily evaluates the severity of heart failure–related symptoms, and certain comorbid conditions may mimic these symptoms, potentially leading to artificially elevated questionnaire scores. Additionally, the relatively low prevalence of certain comorbidities within the study sample may limit the accuracy of conclusions regarding their association with quality of life. Another limitation of the present analysis is the use of the conservative Cook’s distance threshold of 1.0. Although no observation exceeded this value, the application of more stringent criteria, such as 4/n, might have identified patients exerting a moderate influence on the regression results.

Another limitation of this study relates to the hospitalization status of the included patients. Because participants were evaluated during hospitalization, their health-related quality-of-life responses may have been influenced by the acute clinical context, symptom burden, or psychological stress associated with hospital admission. Consequently, HRQoL scores observed in this study may differ from those reported in stable outpatient populations, which may limit the external generalizability of the findings.

Also, another limitation of the present study is the absence of several heart failure–specific variables that may significantly influence health-related quality-of-life outcomes, including heart failure etiology (ischemic vs. non-ischemic), NT-proBNP levels, disease duration, and history of previous hospitalizations. These factors are known to reflect disease severity and clinical trajectory and may therefore influence symptom burden and patient-reported quality-of-life scores. Physical activity levels were assessed using self-reported information, which may introduce recall bias and may not fully reflect the actual level of habitual physical activity.

The relatively small sample size in relation to the number of predictors included in the regression analysis represents an important methodological limitation that may increase the risk of model overfitting. Therefore, the results of the multivariable analysis should be interpreted cautiously and considered primarily hypothesis-generating. Larger studies with more extensive populations are needed to confirm these findings. Additionally, due to the cross-sectional design of the study, causal relationships between clinical characteristics and health-related quality-of-life outcomes cannot be established. The findings should therefore be interpreted as associations rather than causal effects, and longitudinal studies are needed to further clarify the temporal relationships between clinical variables and HRQoL in patients with heart failure. Additionally, multiple statistical comparisons were performed without formal correction, which may increase the risk of type I error. Therefore, the results should be interpreted cautiously. Another important limitation of this study is the absence of treatment-related variables in the multivariable analysis. Guideline-directed medical therapy, including angiotensin receptor–neprilysin inhibitors, beta-blockers, mineralocorticoid receptor antagonists, SGLT2 inhibitors, and diuretics, is known to significantly influence symptom burden and health-related quality-of-life outcomes in patients with heart failure. Variability in treatment optimization among patients may therefore have influenced HRQoL scores in our cohort. Future studies should incorporate detailed pharmacological treatment data in order to better evaluate the relationship between clinical variables, therapy, and quality-of-life outcomes.

Despite these limitations, the study provides several relevant methodological and clinical contributions. The multivariate analytical approach allowed adjustment for multiple comorbidities and potential confounding variables, yielding independent and robust estimates of the associations analyzed. Furthermore, the study incorporated variables that are less frequently addressed in the existing literature. Rather than grouping conditions into broad categories (e.g., “respiratory diseases”), we analyzed specific subtypes such as OSAS, chronic obstructive pulmonary disease, and bronchial asthma, thereby offering a more detailed characterization of the impact of comorbidities on quality of life. In addition, other cardiovascular comorbidities that are less commonly explored from a quality-of-life perspective, such as valvular heart disease, were also included.

## 5. Conclusions

In conclusion, the present analysis identified several conditions associated with a higher quality of life, including atrial fibrillation with electrostimulated cardiac rhythm, type 2 diabetes mellitus, arterial hypertension, chronic hepatitis, and chronic gastritis. In addition, female patients and those with a moderate level of physical activity reported lower MLHFQ scores, indicating better perceived quality of life.

Conversely, several factors were associated with a negative association with quality of life when concomitant with heart failure. These included specific comorbidities such as valvular heart disease, obstructive sleep apnea syndrome, cervical–thoracic–lumbar polydiscopathy, hyperthyroidism, and hepatic steatosis, as well as disease-related characteristics such as higher NYHA functional class and reduced ejection fraction. Furthermore, a progressive decline in quality of life was observed with advancing age.

## Figures and Tables

**Table 1:** Pairwise Comparison Using the Dwass–Richardson–Fligner Test.

Group 1	Group 2	W	*p*
**Preserved**	Mildly Reduced	6.05	<0.01 *
**Preserved**	Reduced	−2.69	0.137
**Mildly Reduced**	Reduced	−5.07	<0.01 *

* W, Dwass–Steel–Critchlow–Fligner test statistic; *p*, *p*-value.

**Table 2:** Descriptive Analysis of MLHFQ Scores.

	MLHFQ Total Score	MLHFQ Physical Subscore	MLHFQ Emotional Subscore
**Mean ± Standard Deviation** **Median [Quartile 1, Quartile 3]**	28.5 ± 10.6	13.8 ± 5.88	-
**Mean ± Standard Deviation** **Median [Quartile 1, Quartile 3]**	-	-	7 [4, 10]

MLHFQ, Minnesota Living with Heart Failure Questionnaire. Data are presented as mean ± standard deviation or median [quartile 1, quartile 3], as appropriate. Continuous variables with normal distribution are presented as mean ± standard deviation (SD), whereas non-normally distributed variables are reported as median and interquartile range [IQR].

**Table 3:** Comparison of the MLHFQ emotional subscore according to clinical variables.

	Median (Yes)	Median (No)	W	*p*
**Diabetes mellitus**	4.5	7	1318	0.033 *
**Valvular heart disease**	7	5	1520	0.102
**Chronic ischemic heart disease**	6	7	992	0.072
**Chronic kidney disease**	5	7	757	0.490
**Respiratory diseases**	7.5	6	1402	0.193
**Gastrointestinal diseases**	7	6	1596	0.263
**Thyroid disorders**	4	7	403	0.299
**Orthopedic diseases**	6	7	861	0.271
**Arterial hypertension**	7	6	1001	0.259
**Sex**	6	7	1610	0.465

* W, Wilcoxon rank-sum test statistic; *p*, *p*-value. Data are presented as medians.

**Table 4:** Kruskal–Wallis test for comparison of the MLHFQ emotional subscore.

Variables	χ^2^	df	*p*
**NYHA functional class**	4.10	2	0.129
**Arterial hypertension**	9.62	2	0.008 *
**Ejection fraction**	1.67	2	0.433
**Activity level**	8.37	2	0.015 *

* χ^2^, chi-square test statistic; df, degrees of freedom; *p*, *p*-value.

## Data Availability

No new data were created or analyzed in this study. Data sharing is not applicable to this article.

## Abbreviations

The following abbreviations are used in this manuscript:

AF	Atrial fibrillation
AF-CHF	Atrial Fibrillation and Congestive Heart Failure
ARNI	Angiotensin receptor–neprilysin inhibitor
BMI	Body mass index
CHF	Congestive heart failure
CHFQ	Chronic Heart Failure Questionnaire
CKD	Chronic kidney disease
CIC	Chronic ischemic cardiomyopathy
COPD	Chronic obstructive pulmonary disease
CS	Clinical summary
DM	Diabetes mellitus
EQ-5D-5L	EuroQol 5-Dimension 5-Level questionnaire
ESC	European Society of Cardiology
GERD	Gastroesophageal reflux disease
GOLD	Global Initiative for Chronic Obstructive Lung Disease
HF	Heart failure
HFpEF	Heart failure with preserved ejection fraction
HRQoL	Health-related quality of life
HTN	Arterial hypertension
KCCQ	Kansas City Cardiomyopathy Questionnaire
KCCQ-CS	Kansas City Cardiomyopathy Questionnaire–Clinical Summary
KCCQ-OS	Kansas City Cardiomyopathy Questionnaire–Overall Summary
LVEF	Left ventricular ejection fraction
MLHFQ	Minnesota Living with Heart Failure Questionnaire
NYHA	New York Heart Association
OSAS	Obstructive sleep apnea syndrome
PM	Electrostimulated cardiac rhythm
SF-36	Short Form 36 Health Survey
SGLT-2	Sodium–glucose cotransporter-2
SR	Sinus rhythm
